# New Imaging Methods for Non-invasive Assessment of Mechanical, Structural, and Biochemical Properties of Human Achilles Tendon: A Mini Review

**DOI:** 10.3389/fphys.2016.00324

**Published:** 2016-07-27

**Authors:** Alexandre Fouré

**Affiliations:** Aix-Marseille Université, Centre National de la Recherche Scientifique, Centre de Résonance Magnétique Biologique et Médicale CRMBM UMR 7339Marseille, France

**Keywords:** ultrasonography, elastography, magnetic resonance imaging, tendon stiffness, tendinopathy

## Abstract

The mechanical properties of tendon play a fundamental role to passively transmit forces from muscle to bone, withstand sudden stretches, and act as a mechanical buffer allowing the muscle to work more efficiently. The use of non-invasive imaging methods for the assessment of human tendon's mechanical, structural, and biochemical properties *in vivo* is relatively young in sports medicine, clinical practice, and basic science. Non-invasive assessment of the tendon properties may enhance the diagnosis of tendon injury and the characterization of recovery treatments. While ultrasonographic imaging is the most popular tool to assess the tendon's structural and indirectly, mechanical properties, ultrasonographic elastography, and ultra-high field magnetic resonance imaging (UHF MRI) have recently emerged as potentially powerful techniques to explore tendon tissues. This paper highlights some methodological cautions associated with conventional ultrasonography and perspectives for *in vivo* human Achilles tendon assessment using ultrasonographic elastography and UHF MRI.

## Introduction

The mechanical properties of tendon are highly involved in muscle tension transmission to the skeleton and in the storage-recoil process of elastic potential energy (Alexander and Bennet-Clark, 1977; Roberts et al., 1997) playing an important role in daily activities and sport practices. Tendons exhibit non-linear viscoelastic behavior (Fung, 1981) which has a direct effect on the efficiency of muscular tension transmission to the skeleton and limits stress on muscle (e.g., buffer effect of tendon to slow down eccentric velocity of muscle contraction in jump landing). Tendon viscoelastic properties are highly influenced by the composition of tendinous tissues (Langberg et al., 2001; Kjaer et al., 2009; Thorpe et al., 2012) and especially collagens, proteoglycans, and water (Kjaer, 2004; Wang, 2006; Connizzo et al., 2013). While it is very difficult to dissociate both elastic and viscous behaviors, it has been shown that the crosslinking of collagens increases the elastic modulus and reduced strain at failure (Thompson and Czernuszka, 1995) whereas several components of the extracellular matrix (Kjaer, 2004), especially water (van der Rijt et al., 2006) and proteoglycans (Yoon and Halper, 2005), can be associated with the viscous behavior observed during assessment *in vitro* (Silver et al., 2002; Gautieri et al., 2012).

**Figure 1:**
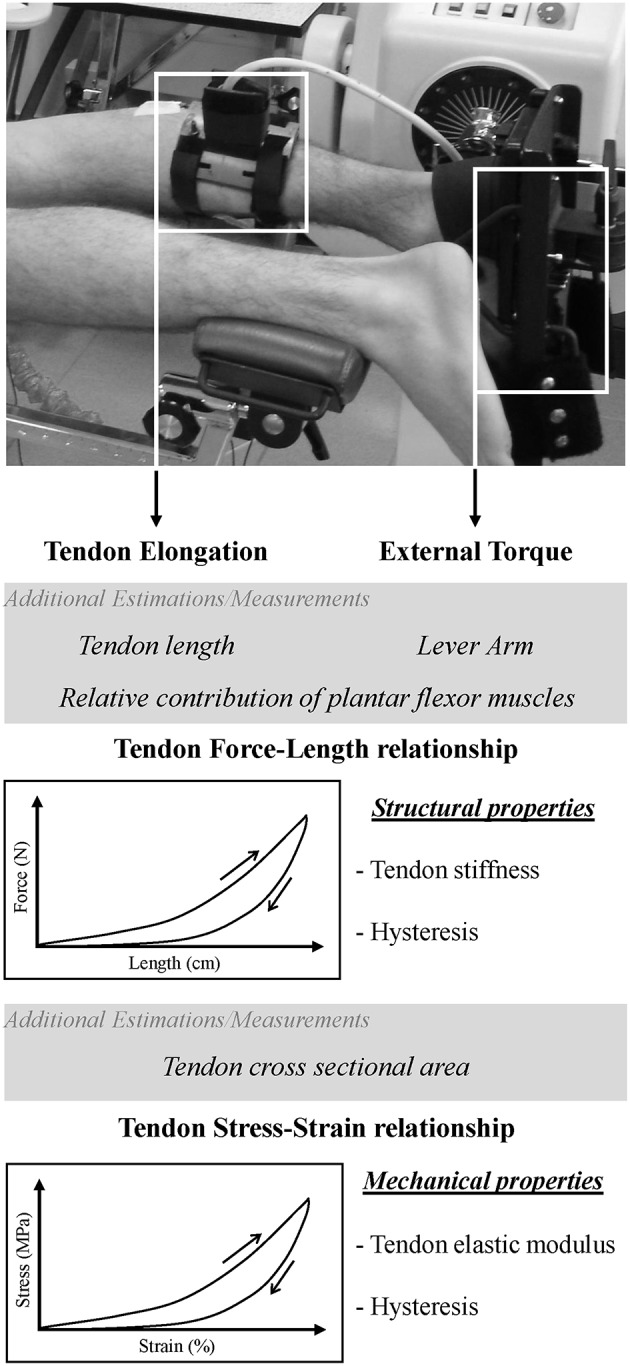
**Experimental position of the subject during assessment of Achilles tendon structural and mechanical properties with ultrasonography and dynamometry**. During passive motion of ankle joint or isometric ramp of plantar flexors contraction, displacement of myotendinous junction is recorded with ultrasonography while external torque is measured with an isokinetic dynamometer. Considering additional measurements of tendon length or estimations of lever arm and the relative contribution of muscles involved in tendon elongation, force-length relationship is established in order to characterize tendon stiffness (structural property, dependent of tendon geometry) and hysteresis in case of loading-unloading cycle. In addition, stress-stain relationship of the tendon can be determined from initial tendon length and tendon cross sectional area. Elastic modulus of tendinous tissues can further be assessed (mechanical property intrinsically related to the tissue, independent of the geometry).

Over the last two decades, ultrasonography has remained the gold standard method to assess tendon structural and mechanical properties non-invasively *in vivo* (Fukashiro et al., 1995; Maganaris and Paul, 2000, 2002; Maganaris, 2002; Magnusson, 2002; Kubo et al., 2002c; Arampatzis et al., 2005). Effects of aging (Magnusson et al., 2003a; Mademli and Arampatzis, 2008; Kubo et al., 2014), gender (Kubo et al., 2003; Magnusson et al., 2007; Westh et al., 2008), rehabilitation (Arya and Kulig, 2010; Geremia et al., 2015), bedrest (Kubo et al., 2000, 2004) or chronic interventions such as training (Kubo et al., 2002a,b; Fouré et al., 2010, 2013) have been abundantly studied. From an experimental point of view, structural and mechanical tendon properties are commonly assessed *in vivo* from the force-elongation and stress-strain relationships (Figure 1) obtained with a constant increase in tension (i.e., loading phase) applied on the tendon due to a passive stretching of muscle tendon unit (Morse et al., 2008) or a controlled isometric contraction (Fouré et al., 2010). Tendon length change is then measured from ultrasound images and synchronized to external torque measured in most cases by a dynamometer (Maganaris and Paul, 2002). In addition, tendon hysteresis can also be determined from the relationship including loading and unloading phases (Maganaris and Paul, 2000). Many methodological strategies associated with the experimental conditions have been reported, discussed and reviewed (Maganaris, 2005; Arampatzis et al., 2008; Finni et al., 2013; Lichtwark et al., 2013; Seynnes et al., 2015). The real time evaluation of tendon length changes used in the non-invasive assessment of tendon structural properties during contraction is a considerable advantage of ultrasound imaging. However, there remain associated methodological issues from the limited spatial coverage of ultrasound probes (e.g., 2D assessment of 3D stain, restricted planar field of view) and the data normalization required to calculate force or stress values from external torque measurements and strain on the basis of tendon elongation. Indeed, most of the published studies chose arbitrary values for tendon cross sectional area (CSA) and slack length (Maganaris, 2002; Magnusson et al., 2003b; Arya and Kulig, 2010) when it is now well known that the Achilles tendon slack length does not correspond to the tendon length when the ankle joint angle is at 90° (Nordez et al., 2010; Hug et al., 2013) and that CSA is not homogeneous along the tendon (Bohm et al., 2015; Lenskjold et al., 2015). Other limitations were previously reviewed in detail (Seynnes et al., 2015). Thus, the mechanical properties of tendinous tissues assessed from stress-strain relationship estimated from external torque measurement and ultrasound imaging can appear inaccurate considering uncontrolled or arbitrary fixed parameters and variability among individuals. To avoid these methodological issues associated with the experimental design and choices in the normalization of the parameters, additional special imaging technologies have been developed to quantitatively assess biomechanical and biochemical properties of tissues.

**Figure 2:**
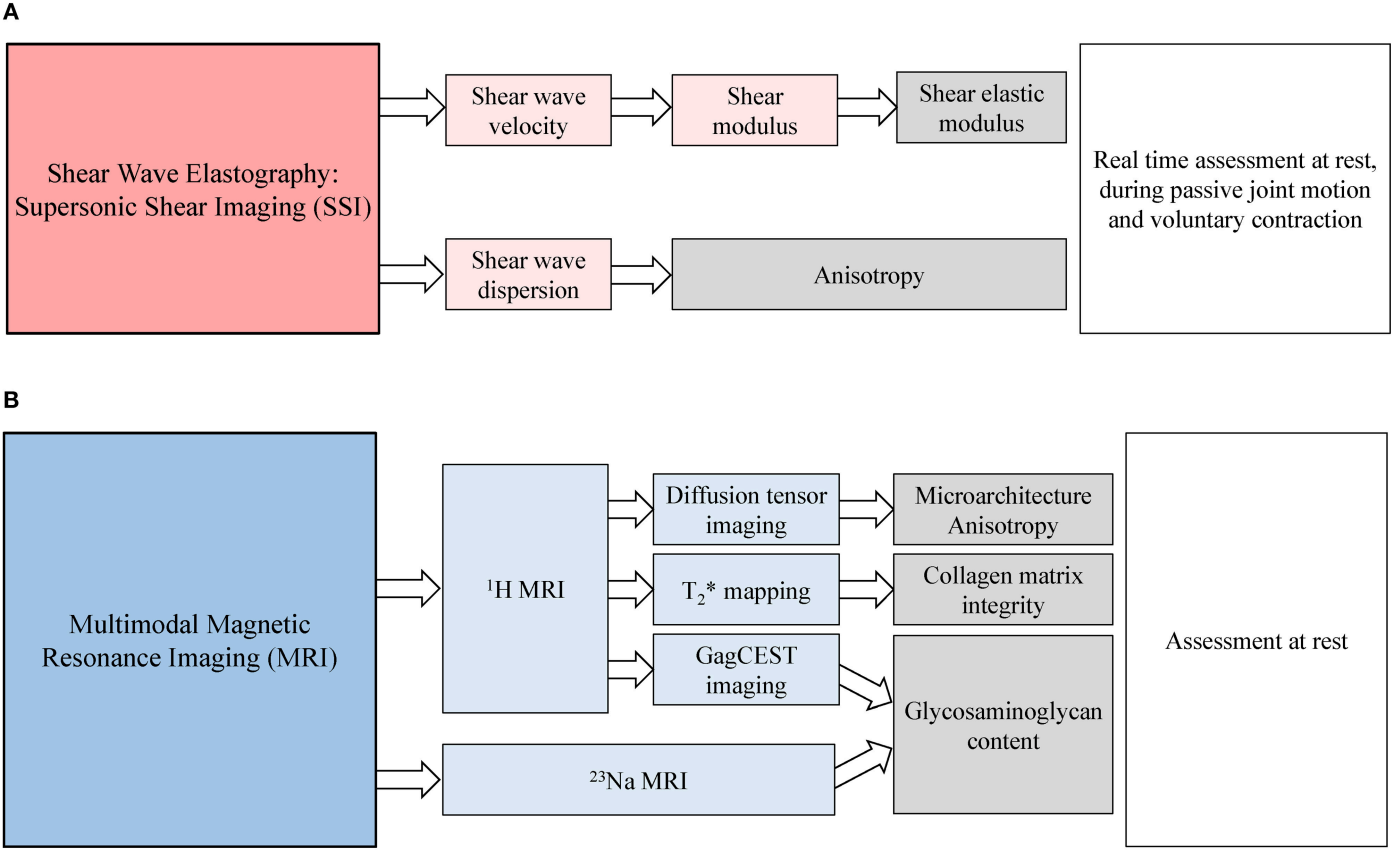
**Schematic representation of emergent imaging techniques for the assessment of tendon biomechanical properties and biochemical composition. (A)** Supersonic Shear Imaging can be used to assess shear elastic modulus and anisotropy of the tendon *via* the analysis of shear wave velocity and dispersion. **(B)** Multimodal Magnetic Resonance Imaging can be used to quantify glycosaminoglycan content, assess collagen matrix integrity and characterize diffusivities of water molecules inside tendon at rest.

The goal of this paper is to highlight the emergence of new imaging methodologies as powerful tools for the non-invasive exploration of tendinous tissues. Although for now elastography is mainly used on skeletal muscle (Bensamoun et al., 2006; Hug et al., 2015), technical evolution of ultrasonographic devices increases applications of elastography on tendon (Helfenstein-Didier et al., 2016). This mini-review is focused on recent developments and applications of ultrasound elastography and ultra-high field magnetic resonance imaging for the non-invasive assessment of tendon biomechanical and biochemical properties (Figure 2).

## Assessment of Achilles tendon biomechanical properties with elastography

Over the last decade, there has been growing evidence that elastography may be a useful tool in detecting subtle changes in musculotendinous mechanical properties that occur early in the course of an injury or disorder.

### Ultrasound elastography

Application of ultrasound elastography on skeletal muscle has been widely developed (Bercoff et al., 2004; Gennisson et al., 2013; Brandenburg et al., 2014). As reported in a recent review, “*supersonic shear wave imaging (SSI) is the current state of the art in ultrasound elastography”* (Hug et al., 2015). This latter technology consists in applying a stress on soft tissue through the acoustic radiation force of a long burst of focused ultrasound pulses (i.e., ultrasound push beams) producing a shear wave observed *via* high frame pulse-echo ultrasound imaging. The time shift of the ultrasound echo is then used to measure deformation associated with the displacement of the shear wave in the tissue. Shear wave elastography is a quantitative method to measure the shear wave velocity to estimate the localized elastic properties of tissue *in vivo* (Bercoff et al., 2004; Brandenburg et al., 2014). Considering tissue as elastic and homogeneous, the shear modulus (μ, kPa) is calculated using the equation: μ = ρ*V*^2^, where ρ is the density of the tissue (kg.m^−3^) and *V* the shear wave velocity (m.s^−1^) (Bercoff et al., 2004; Helfenstein-Didier et al., 2016). Considering isotropic locally homogeneous and quasi-incompressible biological tissues, Young's modulus (*E*) can be estimated from the shear modulus using the following equation: *E* ≈ *3*μ (Bercoff et al., 2004; Hug et al., 2015). While many studies have recently assessed muscle mechanical properties with SSI (Lacourpaille et al., 2014; Hug et al., 2015; Le Sant et al., 2015), this method was also used to determine the slack length of Achilles tendon during passive stretches of the ankle (Hug et al., 2013). Although measurements saturate at relatively low tension levels in the latter study (~20° in plantar flexion during passive stretching) due to the high shear wave speed observed in stiff tissue such as tendon, feasibility, and accuracy of the slack length measurement with SSI was demonstrated (Hug et al., 2013). Given that muscle and tendon slack lengths correspond to different ankle angles, the latter study also shed light on the complex interaction between muscles and Achilles tendon during passive motion of lower limbs.

In the last 5 years, a growing interest in tendon mechanical properties assessment with SSI can be observed. For instance, differences in shear wave velocities have been highlighted among musculotendinous structures (i.e., tendon, aponeurosis, muscle) and according to age (Slane et al., 2015, 2016; Turan et al., 2015). Arda et al. (2011) showed that Young's modulus at rest is higher in Achilles tendon (52 ± 25 kPa in transverse plane and 74 ± 46 kPa in longitudinal plane) as compared to the gastrocnemius muscle (11 ± 4 kPa in longitudinal plane). It is noteworthy that a high inter-individual variability was shown (Arda et al., 2011) and shear wave elastography measurements were dependent on imaging plane (Chino et al., 2015). From a methodological point of view, shear wave elastography in soft tissues such as muscle and tendon must be performed with the lightest transducer pressure (Kot et al., 2012). In addition, both transducer positioning and limb posture have to be carefully checked to limit spatial variations in Achilles tendon shear wave speed (DeWall et al., 2014). Aubry et al. (2013) found an effect of ankle joint angle on shear wave propagation velocity. In the latter study, Achilles tendon anisotropy was calculated on the basis of the difference in the shear wave speed determined in the axial and the longitudinal planes (Aubry et al., 2013). The relative anisotropy coefficient and elastic modulus of Achilles tendon were increased when the ankle was dorsiflexed (Aubry et al., 2013). Brum et al. (2014) assessed the elastic anisotropy of the human Achilles tendon using shear wave dispersion analysis. In this study, shear wave velocity dispersion was measured in both axial and longitudinal planes to the Achilles tendon fiber orientation. Given that the shear wavelength in the longitudinal direction is five times larger than the mean tendon thickness, the wave propagation is guided along the tendon by successive reflections at the tendon boundaries (Brum et al., 2014). Hence, the use of a specific model was needed to characterize tendon viscoelastic properties taking into account the latter phenomenon. On the basis of the shear wave dispersion analysis developed by Brum et al. (2014), Helfenstein-Didier et al. (2016) found that Achilles tendon shear modulus increases with passive dorsiflexion. Regardless of ankle angle, the shear modulus was significantly higher in the proximal region of the Achilles tendon as compared to the more distal one. Very good reproducibility results were reported with coefficients of variation lower than 1% and shear modulus values determined with the shear wave dispersion analysis and conventional shear wave elastography technique were highly correlated (*r* = 0.844, *P* < 0.001). This indicates that SSI can be used to compare tendon mechanical properties determined from shear modulus and then shear elastic modulus across populations and could have a clinical relevance in tendinopathy (Helfenstein-Didier et al., 2016). It is noteworthy that the assumption associated to the SSI technique of an elastic and homogeneous medium is not necessarily entirely correct in the tendon. In fact, the anisotropic characteristics and inter-individual variabilities in microarchitecture of the tendon could limit interpretations when comparing healthy individuals or assessing the effects of interventions with SSI. In addition, measurement of shear elastic modulus with SSI is based on a constant tendon density which can be different in healthy and pathologic tendons. Indeed, it was clearly shown that tendinopathies can induce changes in the structural organization and biochemical composition of tendinous tissues (de Mos et al., 2007; Pingel et al., 2014) leading to a potential inaccuracy in shear elastic modulus assessment. Nevertheless, relevant clinical assessment of tendon disorders assessed with SSI have already been reported in a recent review (Klauser et al., 2014). For instance, lower tendon stiffness was found in patient with torn Achilles tendon as compared to healthy subjects (Chen et al., 2013) suggesting that shear wave elastography provides relevant biomechanical information for Achilles tendon function assessment. In addition, a lower shear wave velocity was found in stretched Achilles tendon of patients with tendinopathy as compared to healthy control subjects whereas no significant difference was reported in Achilles tendon anisotropy (Aubry et al., 2015).

Furthermore, 3D ultrafast ultrasound imaging for the 3D mapping of stiffness, tissue motion, and flow in humans was recently demonstrated *in vivo* as a future new clinical application of ultrasound with reduced intra- and inter-observer variability (Provost et al., 2014). On that basis, it can be expected that methodological developments for 3D assessment of mechanical properties in soft tissues with SSI will continue to progress. However, for now, the 3D exploration of musculoskeletal system with shear wave elastography remains associated with nuclear magnetic resonance techniques (Muthupillai et al., 1995).

### Magnetic resonance elastography

In comparison to ultrasound elastography, magnetic resonance elastography (MRE) has the advantages of full 3D acquisition and can explore deep muscles with large spatial coverage in a well-defined and reproducible coordinate system (Gennisson et al., 2010). MRE typically uses vibrations of a single frequency within the audio frequency range. The shear waves are generated by an electro-mechanical transducer on the surface of the skin and the tissue motion is measured using MRI technique called phase-contrast MRI (Muthupillai et al., 1995). MRE allows assessment of mechanical properties of soft tissues (Mariappan et al., 2010) such as skeletal muscle (Dresner et al., 2001; Bensamoun et al., 2006). MRE has been used to investigate non-invasively lower limb skeletal muscles mechanical properties and detect abnormalities in patients with neuromuscular disorders as compared to matched control subjects (Basford et al., 2002). However, in early studies on skeletal muscle, neither viscosity nor anisotropy were taken into account to assess mechanical properties. Subsequent MRE studies demonstrated the high anisotropy of the muscle tissue (Papazoglou et al., 2005). Recently, multi-frequency MRE and rheological models were used to assess the viscoelastic shear properties of thigh muscles in passive condition *in vivo* (Chakouch et al., 2016). So far, MRE has only been used on skeletal muscle and no exploration on tendon has been performed *in vivo*. Indeed, very stiff tissues such as tendon (in comparison to skeletal muscle) require much higher vibration frequencies for mechanical properties assessment with MRE. Current MRI scanners do not have gradient hardware that is capable of encoding wave motion at such high frequencies (Mariappan et al., 2010). These limitations may be addressed in the future with specialized hardware solutions and development of ultra-high field (UHF) MRI clinical scanners with special high-speed imaging techniques (Glaser et al., 2006).

Considering that the intrinsic mechanical properties of the tendinous tissues are closely related to the tendon composition, UHF MRI appears a powerful imaging technique to assess microstructural and biochemical parameters closely linked to the viscoelastic behavior of the Achilles tendon; meanwhile, technological development is still necessary for the direct assessment of the tendon mechanical properties with MRE.

## Assessment of Achilles tendon biochemical and structural properties with MRI

Magnetic resonance imaging allows an accurate assessment of biochemical composition and microstructure of musculoskeletal tissues. However, visualization of the tendon remains difficult due to a very short transverse relaxation time (i.e., T_2_ < 1.5 ms) leading to a partial or total disappearance of signal in the tendon with relatively long echo time used in conventional clinical MRI sequences (Gatehouse and Bydder, 2003). Thus, special MR sequences are required to acquire signal from the tendon. The most frequently used sequence in the recent studies consisted in a quantitative imaging of tendon using ultrashort echo time (UTE; Robson et al., 2004; Juras et al., 2012b) and variable echo time sequences (Song and Wehrli, 1998; Juras et al., 2013b).

Furthermore, improved signal to noise ratio (SNR) in emerging 7 Tesla (7T) whole body MRI scanners—SNR being proportional to the field strength—provides opportunities for easier examinations of musculoskeletal structures (Trattnig et al., 2015) and especially Achilles tendon (Trattnig et al., 2012). Hence, many studies are now using 7T-MRI to explore tendon structure and quantitatively assess structural and biochemical properties *in vivo* (Trattnig et al., 2012). For instance, Han et al. succeeded in using high-resolution 3D UTE sequence to visualize the Achilles tendon microstructure in human healthy volunteers (Han et al., 2014).

### Tendon relaxation constants

Quantitative MRI is widely used to characterize potential alteration of musculoskeletal tissues. Change in T_2_^*^ relaxation time is commonly assessed in skeletal muscle to quantify the effects of edema/inflammation processes associated with acute injury (Fouré et al., 2015b) or muscle disease (Arpan et al., 2013).

Despite the very short transverse relaxation time of the tendon, T_2_^*^ is the most popular relaxation constant used to detect potential collagen matrix alteration. Using variable echo time sequence, the bi-exponential T_2_^*^ signal decay has been analyzed in healthy subjects and patients with tendinopathies (Juras et al., 2012b, 2013b). A strong correlation between clinical score and the short component T_2_^*^ of pathologic tendon was demonstrated at 3T (Juras et al., 2013b). It is noteworthy that the increased SNR at 7T can provide higher accuracy of T_2_^*^ calculation as compared to 3T (Juras et al., 2012b). Although the short component of T_2_^*^ was shown to be a robust and promising biomarker of tendon structural alterations (Chang et al., 2016), other parameters such as glycosaminoglycan content appear to be more specific to the changes in biochemical properties of injured or pathologic tendon.

The T_2_ assessment of supraspinatus tendon was shown to be reproducible (Anz et al., 2014) but it was only assessed on healthy volunteers and no data are available to check the sensitivity of this parameters to structural and/or biochemical changes in patients with tendinopathies and tendon injuries.

Another constant associated to both transverse and longitudinal relaxation times of the tissue (T_1_ρ) is also used to assess musculoskeletal tissues (Wang and Regatte, 2015) and especially cartilage (Regatte et al., 2003). However, only one study has assessed Achilles tendon T_1_ρ of cadaveric specimens and healthy control subjects using a 2D UTE T_1_ρ sequence (Du et al., 2010). For now, T_1_ρ is mostly used in basic sciences and does not yet have widespread clinical use.

### Glycosaminoglycans content assessment

The detection of biochemical changes can help the early diagnosis of tendinopathy (Samiric et al., 2009). An increased amount of proteoglycan content in the extracellular matrix was reported in human pathologic tendons (Fu et al., 2007; Parkinson et al., 2010). The sulfate and carboxyl groups associated with glycosaminoglycans (GAG) provide proteoglycans with a net negative charge, attracting molecules with positive charge such as sodium ions. Thus, a strong correlation was reported between GAG content and sodium MRI at UHF in *ex vivo* tendon (Juras et al., 2013a). The increased GAG content in the tendon was also correlated with the Victorian Institute Sport Assessment (VISA) score (Attia et al., 2014), a widely used outcome measure of functional status and pain level. Expression of several proteoglycans typically associated with GAGs such as decorin, versican, and aggrecan were found to be higher in pathologic patellar tendon as compared to controls (Attia et al., 2014).

Sodium MRI is an imaging technique based on detection of ^23^Na nuclei used to quantify sodium content in biological tissues which can provide an indirect quantification of tendon GAG content. A higher tendon sodium SNR was also reported in patients with tendinopathy as compared to healthy control (Juras et al., 2012a). Sodium MRI appears as a powerful and non-invasive method to detect early biochemical changes in tendinopathy. However, sodium imaging requires an MR system with multinuclear capabilities and dedicated sodium antenna coils. To bypass this issue, chemical exchange saturation transfer (CEST) can be used to assess GAG content (GagCEST) in the tendon. The method was first developed in cartilage (Schmitt et al., 2011) and provided an index of GAG content on the basis of chemical exchange between bulk water protons and protons bound to solutes (Guivel-Scharen et al., 1998). This method is emerging as a relevant alternative to sodium MRI but requires complex image post-processing. In addition, for accurate quantification of GagCEST effects, it is essential to account for inhomogeneities of the static magnetic field B_0_ and radiofrequency field B_1_. Nevertheless, reproducible measurements in knee cartilage of healthy volunteers have been recently provided (Schreiner et al., 2016).

So far, only one study has assessed Achilles tendon biochemical properties with multimodal MRI (i.e., T_2_^*^ mapping, sodium MRI and GagCEST; Juras et al., 2015). This study assessed effects of ciprofloxacin intake on Achilles tendon properties of seven healthy males. It was previously shown that fluoroquinolones such as ciprofloxacin can increase risk of tendon injuries (Stephenson et al., 2013). While no significant change in morphology and collagen matrix was detected with T_2_^*^ mapping, a decrease in GAG content was seen 10 days after ciprofloxacin intake using both sodium MRI and GagCEST. This biochemical change was not associated with clinical symptoms of tendon injury. Therefore, multimodal MRI could potentially be used to detect onset of abnormal change in tendon GAG content representing one of the early stages of tendinopathy.

It was previously shown that tendon stiffness assessed with ultrasonography and dynamometry is decreased in patients with tendinopathies (Arya and Kulig, 2010). In addition, changes in extracellular matrix highly influence tendon function (Kjaer, 2004). Given that stiffness is a structural characteristic of the tendon, dependent on the intrinsic mechanical properties of the tissues (i.e., related to the tendon biochemical composition) and the microarchitecture of the tendon, it appears relevant to concomitantly assess tendon biochemical and microarchitectural properties of the tendon.

### Microarchitecture characterization

Diffusion tensor imaging (DTI) is sensitive to changes in the microstructural architecture of biological tissues. Cell membranes and other solid structures restrict water diffusion leading to anisotropic diffusion. The integrity of tissues is then assessed by the predominant direction, intensity and isotropic characteristics of water diffusion within the biological structure. For instance, DTI is used in skeletal muscle to determine potential exercise-induced structural alterations (Fouré et al., 2015a) and potential changes in muscle architecture (i.e., fiber length and pennation angle) with muscle fiber tractography (Cotten et al., 2015). For now, only a few studies have assessed anisotropy/microarchitecture of the tendon (Momot et al., 2010) in animals (Wellen et al., 2005; Helmer et al., 2006; Gupta et al., 2010) and in humans (Sarman et al., 2015). Since tendon has a short transverse relaxation time, methodological developments are needed to obtain shorter echo time than those available on conventional clinical scanners. New methodologies have recently been presented in the *ISMRM* annual meeting (He et al., 2016; Ma et al., 2016). Although methodological and experimental issues such as dependence of tendon fibers orientation in the static magnetic field (i.e., magic angle effect) have to be resolved, there is likely to be additional methodological developments for tendon microarchitecture assessment with UHF MRI and growing interest of the MR community for tendon assessment.

## Conclusion

Non-invasive imaging methods for the assessment of human Achilles tendon *in vivo* have been widely adopted in the two last decades. Democratization in the use of ultrasonographic devices and technical developments in both ultrasound and MRI widen the perspectives for tendon assessment in the clinical context and for basic science. For now, ultrasonography is the most popular tool to assess tendon structural properties. However, the development of elastography based on ultrasound and MRI appears complementary in assessing tendon biomechanical and biochemical properties. While ultrasound elastography allows local assessment of the tendon in real-time and potentially during passive (i.e., passive joint motion) or active (i.e., muscle contraction) stretch, multimodal MRI can accurately assess tendon structural and biochemical properties in three dimensions at rest. Further studies associating quantitative MRI and elastography are needed in order to assess non-invasively the mechanical, structural and biochemical properties of the Achilles tendon and lead to clinical applications for diagnosis, prognosis and follow-up of tendinopathies and tendon injuries.

## Author contributions

AF has designed, written, and approved this manuscript.

### Conflict of interest statement

The author declares that the research was conducted in the absence of any commercial or financial relationships that could be construed as a potential conflict of interest.
